# Multi-species eDNA as a screening tool to facilitate early detection and eradication of aquatic invasive species in large water bodies

**DOI:** 10.1038/s41598-025-19083-7

**Published:** 2025-09-29

**Authors:** Rebecca L. Flitcroft, Brooke E. Penaluna, Laura L. Hauck, Jay W. Munyon, James M. Capurso

**Affiliations:** 1 USDA Forest Service, Pacific Northwest Research Station, Corvallis, OR 97331 USA; 2 USDA Forest Service, Pacific Northwest Regional Office, 97204 Portland, OR USA

**Keywords:** Aquatic invasive species, Monitoring, Multigene eDNA barcoding, Multi-species eDNA, Water, Genetics, Computational biology and bioinformatics, Biodiversity

## Abstract

Aquatic invasive species can devastate native biodiversity and human water infrastructure. Effective eradication relies on early detection. However, commonly used visual surveys are ineffective for detection of small populations of submerged invasive species in large water bodies. Here, we explored detection of invasive aquatic plants, animals (vertebrate and invertebrate), and pathogens using 10 environmental DNA (eDNA) water sampling events every two weeks between June and October, 2018, informing ideal sampling times for long-term early-detection monitoring. The highest number of species detections across taxa were found using 6 replicates in late August and early September. Detections varied by taxon, with the most detections for fishes, followed by invertebrates, amphibians, and submerged plants. All expected species were detected with eDNA except for three terrestrial and emergent riparian plants. Reservoirs had the most consistent presence of AIS, suggesting that those systems and aquatic communities may be susceptible to new invasions. AIS detections occurred across more sites and water bodies than had been previously documented which provided evidence of silent invasions by species such as crayfishes, mollusks, and plants. We offer a framework for interpreting management response to low-read counts from multispecies eDNA sampling that balances interpretation of results with the cost of management responses.

**Supplementary Information:**

The online version contains supplementary material available at 10.1038/s41598-025-19083-7.

## Introduction

Public lands in the western United States and elsewhere are managed for ecological sustainability and the well-being of local communities^1^. An important part of this mandate is the conservation, restoration, and recovery of freshwater habitats that support native aquatic species. One element of this work is vigilance in the detection and eradication of aquatic invasive species (AIS) that have the potential to compromise local habitats, affecting aquatic and riparian ecosystems. Freshwater biodiversity is declining at a faster rate than in either terrestrial or marine environments^2,3^, and competition with, and predation by, AIS is a contributing factor^4,5^. Additionally, as temperature and precipitation changes alter the characteristics of environmental niches, changes in habitat suitability may benefit invasive over native species^6^. AIS are known to have devastating ecological^7^ and economic^8^ impacts if they are allowed to develop strongholds outside their native range, making early detection of these species critical. For example, invasive Zebra Mussels (*Dreissena polymorpha*) cover streambeds, smothering native species, and may completely clog water intake pipes at municipal water and hydropower sites^8^. Thus, monitoring for new invaders is both an immediate and ongoing management priority^9,10^, and these threats lead to real-world challenges as staff and budgets are limited. Further, initial infestation by AIS are difficult to detect leading to development of established populations before managers are aware of the threat.

In freshwater settings, the primary agents of introduction and spread for aquatic invasive species are the boots and boats of recreationalists^11^. This means that some of the highest-risk locations for potential invasion by AIS are the places most frequented by anglers, boaters, and other aquatic recreationists. These navigable-water environments have areas with depths, and/or water flow velocities, that impair visual inspection surveys for identification of submerged invasive species. Ineffective surveys compromise early detection of invasive species, which is crucial for eradication before populations become established in newly colonized environments^12^.

A more sensitive method to detect species in deep or fast water environments is needed to facilitate early detection of AIS. The detection of species-specific DNA in water, or environmental DNA (eDNA), has proven to be an effective tool for detecting early invasion by single species (e.g., Quagga Mussels *Dreissena rostriformis*^13^, *Dreissena* spp.^14^, Zebra Mussels^15^. However, most managers are interested in a diverse array of potential invaders including plants, animals, and pathogens. Multi-species detections using eDNA have been accomplished via “eDNA metabarcoding” where eDNA fragments of many species are amplified, sequenced and then bioinformatically identified^16–18^. This method has been applied to screening species of conservation concern (animals^19,20^; as well as terrestrial and aquatic plants^21,22^, thus, eDNA metabarcoding could be useful as a screening tool for early detection, and potentially early eradication, of a suite of both plant and animal AIS [e.g.^23^]. The advantages of this approach over single-species methods (e.g., quantitative polymerase chain reaction, qPCR or digital droplet PCR, ddPCR) are that it reduces false positives by definitively identifying amplicons rather than relying on qPCR assay specificity, allows for the screening of multiple species from a single water sample, and, when paired with the enormous capacity of modern DNA sequencers, captures both presence information and genetic population diversity at the same time^18,23–27^. Although parallel sequencers are powerful, fully understanding the phenomenon of index/barcode swapping^28,29^ and developing a consistent method to mediate index swaps in eDNA metabarcoding datasets without losing sensitivity is an important consideration.

Our goal was to evaluate how detections of plant and animal species changed over the summer growing season to identify the most effective time for monitoring designs to target large water bodies such as lakes, rivers, and reservoirs. Recent work has shown that eDNA of AIS peaks during different seasons owing to different life histories of taxa^22–24^, and we aimed to build on that work to find an optimal timeframe where managers could focus their sampling effort by maximizing detections for multiple AIS. We hypothesized that detections of most species might occur when flows are lower, as higher discharge dilutes eDNA concentrations^30^. The technical capacity of multigene eDNA metabarcoding to detect a large number of taxa, including fishes, amphibians, crayfishes, bivalves, pathogens, and bryozoans, as well as plants that are riparian, have emergent stems/flowers, or are submerged, in a single array makes this a useful preliminary screening tool for the early detection of invasive species. To facilitate the management response to eDNA-based monitoring for novel aquatic invasive species in large water bodies, we developed a framework for interpreting eDNA metabarcoding results, with associated monitoring and management recommendations based on eDNA metabarcoding detections. We propose that our framework can inform eDNA strategies (e.g.^28^, and management decisions^31^ for multispecies communities of AIS. We demonstrated the utility of this framework through evaluation and follow-up sampling of a low-eDNA detection of a nonnative freshwater mussel.

## Results

**Fig. 1:**
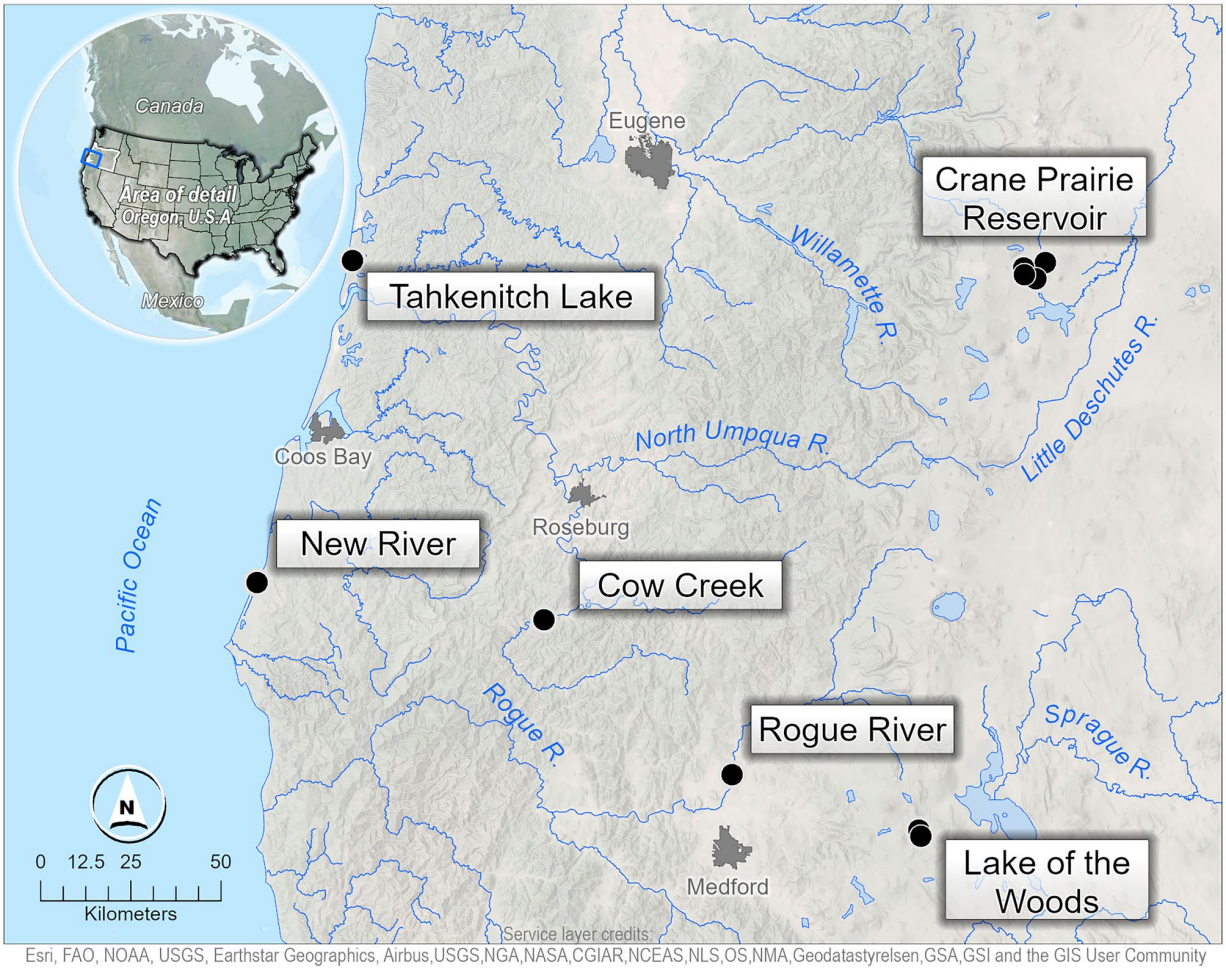
Six study locations were selected in the state of Oregon, USA, to represent three types of large water bodies: natural lakes, reservoirs, and rivers.

To answer our research question and test our framework, we developed metabarcoding primers for the focal species of interest (Table 1) including: 20 primers targeting invasive animal species, 18 primers targeting invasive plants and algae, 2 primer sets targeting pathogens, and 7 universal metabarcoding primers targeting a wide range of organisms (Primer sequences available in Table S1, additional details in Primer Development section of eDNA Laboratory Methods). We conducted field sampling in three navigable rivers, two lakes and one reservoir, encompassing both moist-wet/low-elevation environments and dry/high-elevation environments in Oregon, USA. Sampling was conducted bi-weekly from June through October 2018 (Fig. 1 - see Methods for more information) for a total of 10 sampling events per site.

**Table 1:** Metabarcoding data primers were developed for the focal species of interest identified by forest managers in Oregon.

Common Name	Scientific Name	Taxon	Growth habitat (plants)
American Bullfrog	*Lithobates catesbeianus/Rana catesbeiana*	Amphibian	
Nutria	*Myocastor coypus*	Mammal	
Rusty Crayfish	*Faxonius rusticus*	Decapod	
unknown *Faxonius* crayfish	*Faxonius* spp.	Decapod	
Ringed Crayfish	*Faxonius neglectus*	Decapod	
Northern Crayfish	*Faxonius virilis*	Decapod	
Red Swamp Crayfish	*Procambarus clarkii*	Decapod	
Bullhead	*Ameiurus* spp.	Fish	
Sunfish	*Lepomis* spp.	Fish	
Largemouth Bass	*Micropterus nigricans*	Fish	
Chinese Mystery Snail	*Cipangopaludina chinensis*	Mollusc	
Freshwater Golden Clam	*Corbicula* spp.	Mollusc	
Big-eared Radix	*Radix auricularia*	Mollusc	
Zebra Mussel	*Dreissena polymorpha*	Mollusc	
Quagga Mussel	*Dreissena rostriformis*	Mollusc	
New Zealand Mudsnail	*Potamopyrgus antipodarum*	Mollusc	
Amphibian Chytrid Fungus	*Batrachochytrium dendrobatidis*	Pathogen	
Magnificent Bryozoan	*Pectinatella magnifica*	Bryozoan	
Giant Reed	*Arundo donax*	Plant	Riparian
Yellow Flag	*Iris pseudacorus*	Plant	Riparian
Garden Loosestrife	*Lysimachia vulgaris*	Plant	Riparian
Purple Loosestrife	*Lythrum salicaria*	Plant	Riparian
Reed Canary Grass	*Phalaris arundinacea*	Plant	Riparian
Common Reed	*Pragmites australis*	Plant	Riparian
Flowering Rush	*Butomus umbellatus*	Plant	Emergent stems/flowers
Water Primrose	*Ludwigia* spp.	Plant	Emergent stems/flowers
Yellow Floating Heart	*Nymphoides peltata*	Plant	Emergent stems/flowers
Brazilian Elodea	*Egeria densa*	Plant	Submerged
Water Thyme	*Hydrilla verticillata*	Plant	Submerged
Water Milfoil	*Myriophyllum* spp.	Plant	Submerged
Pondweed (non-native)	*Potamogeton* spp.	Plant	Submerged
Curly-leaf Pondweed	*Potamogeton crispus*	Plant	Submerged
Sago Pondweed	*Stuckenia pectinata*	Plant	Submerged
Didymo	*Didymosphenia geminata*	Plant	Submerged

### Detections of taxa by eDNA

The NovaSeq run produced > 489 million (M) read pairs, of which > 281 M passed filtering criteria and were assigned to a sample and primer of origin, with ~ 148 M read pairs classified to a taxon. Results of our eDNA screening by location, and previously documented presence/absence of species of interest, are summarized in Table 2. We detected both plant, and animal, taxa in the same eDNA sample.

**Table 2:**
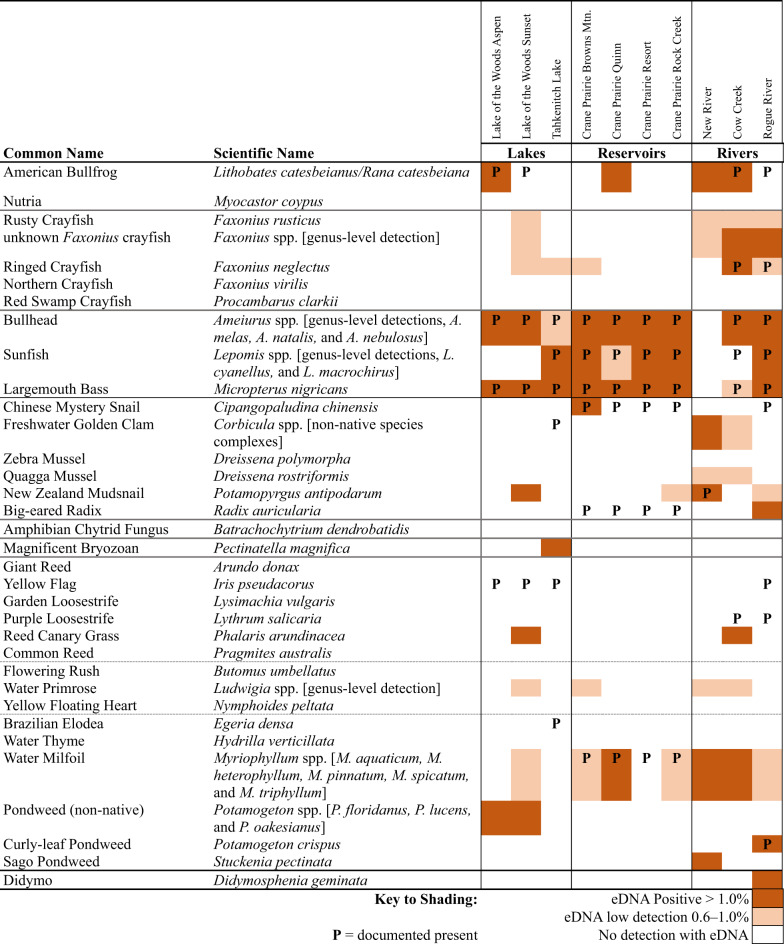
Positive and low detections of focal species at sites located in rivers, natural lakes and reservoirs in Oregon, USA.

Classification of detections as positive, low or non-detected, is linked to the number of classified reads tallied by organism, primer, and sample. Our metabarcoding data present with a long tail distribution of low tallied reads per taxon per site, and some of these are the result of barcode swapping. The sum of the reads in the long tail accounts for less than 1% of the whole dataset, and above that threshold we consider the reads a positive detection. Low detections are defined as being in the bottom 0.6–1.0% of all reads tallied by organism, primer, and sample. All combinations representing < 0.6% at the end of the long tail are attributed to barcode swaps, and are removed from the dataset. More interpretation of these classifications is presented in the “Defining a Detection” section of the Discussion.

#### Vertebrates

**Fig. 2:**
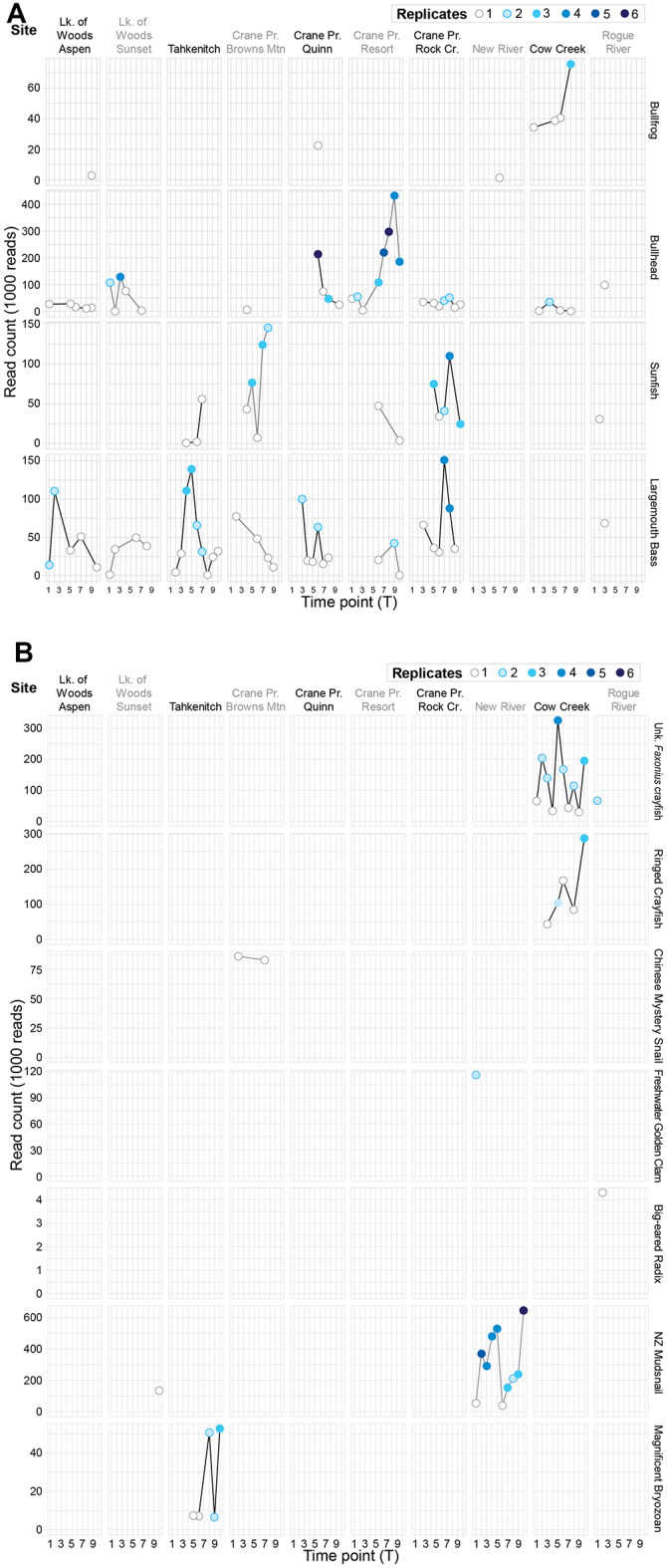

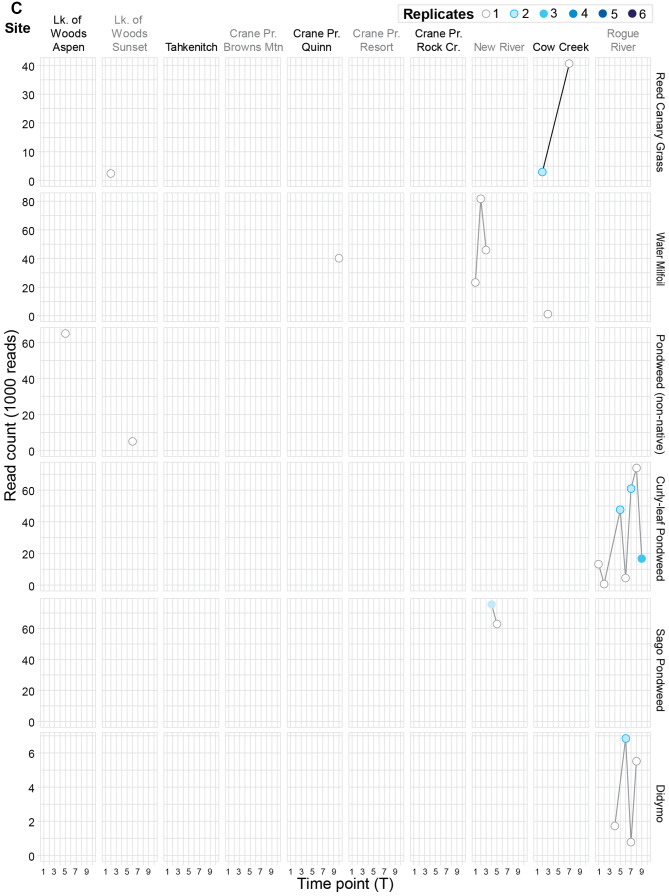
Positive species detections by the number of replicates (6 taken at every sample site/event) varied by location within and among sites over time. (**A**) Positive vertebrate detections included bullfrog and teleost fishes; (**B**) positive invertebrate detections included crayfish, snail, clams, and bryozoan; and (**C**) positive plant detections were generally fully submerged species.

Teleosts, including sunfish (*Lepomis* spp.), bullhead (*Ameiurus* spp.), and Largemouth Bass (*Micropterus nigricans*), had eDNA detections at every location except New River (Fig. 2A; Table 2). Positive fish detections coincided with the documented presence of these species, except at Cow Creek where sunfish were not detected using eDNA (Table 2), and at three locations where eDNA detections were low (Tahkenitch Lake for bullhead, Crane Prairie Quinn for sunfish, and Cow Creek for Largemouth Bass). Across teleosts, positive detections were most often found in one to three of six replicates taken at each site (81 of 88). Five of the seven samples with detections in four or more replicates were for bullhead at Crane Prairie Reservoir. The remaining two samples with four detections were of Largemouth Bass, also at Crane Prairie Reservoir (Fig. 2A).

American Bullfrog (*Lithobates catesbeianus/Rana catesbeiana*) had positive eDNA detections at four locations (Fig. 2A; Table 2), including two detections from previously undocumented occurrences at Crane Prairie Reservoir and New River. Bullfrogs are documented at one other Lake of the Woods site, and in the Rogue River, where they were not detected using eDNA. At Cow Creek, they were detected in multiple time periods, and for the latest detection, were found in three replicates.

#### Invertebrates

Compared to teleost fish, there were fewer positive detections of invertebrate animals by eDNA associated with sites that have documented presence of the invasive species (Table 2). Most crayfish detections were at low-eDNA levels. However, positive detections were found at the genus level for an unknown *Faxonius* crayfish in Cow Creek and the Rogue River (Table 2). Ringed Crayfish (*Faxonius neglectus*) were previously documented and positively detected using eDNA in Cow Creek. In the Rogue River, this species was previously documented but was detected only at low-eDNA levels. Across all target crayfish, positive detections in more than 3 of 6 replicates were uncommon and were found only in unknown *Faxonius* crayfish at Cow Creek (Fig. 2B).

Chinese Mystery Snail (*Cipangopaludina chinensis*) has documented presence at Crane Prairie Reservoir and on the Rogue River, but was positively detected by eDNA at only one sampling site (Browns Mtn at Craine Prairie Reservoir) (Table 2). Freshwater Golden Clam (*Corbicula* spp.) was not detected in Tahkenitch Lake where it has been documented as present, but an undocumented lineage of Freshwater Golden Clam was found with eDNA at New River (and at a low-detection level at Cow Creek). Signal from a mussel was detected at Tahkenitch Lake with the closest match being *Lasmigona costata*, a species native to the southeastern United States, suggesting that this may be an invasive mussel. New Zealand Mudsnail (*Potamopyrgus antipodarum*) was detected at two sites, one with prior documentation (at New River), but also in Lake of the Woods Sunset where it had not previously been documented. Two additional low-eDNA detections for this snail were in Crane Prairie Resort and Rogue River. Big-eared Radix (*Radix auricularia*) was documented as present in Crane Prairie, but was not detected with eDNA. However, eDNA did detect this species at a previously unknown location on the Rogue River (Table 2). Magnificent Bryozoan (*Pectinatella magnifica*) was detected using eDNA in Tahkenitch Lake, where it had not previously been documented (Table 2). Among invertebrates, positive detections occurring in more than 3 replicates were found for New Zealand Mudsnail in New River, where 5 of the 10 sampling events across the sampling season found positive detections in four or more replicates; and for unknown *Faxonius* crayfish at Cow Creek (Fig. 2B).

#### Plants and algae

Submerged aquatic plants with known presence were identified using eDNA at positive and low-eDNA levels in four of six locations (Table 2; Fig. 2C). Detections of previously undocumented submerged aquatic plants and algae included Sago Pondweed (*Stuckenia pectinata*) in New River, *Potamogeton* spp. in both Lake of the Woods sample locations, and Didymo (*Didymosphenia geminata*) on the Rogue River (Table 2). Reed Canary Grass (*Phalaris arundinacea*) was the only riparian plant detected by eDNA. Yellow Flag Iris (*Iris pseudacorus*) and Purple Loosestrife (*Lythrum salicaria*) have known presence in several of our study locations but were not detected with eDNA. Water Primrose (*Ludwigia* spp.) is an emergent aquatic plant that was detected at low-eDNA levels in several sites in this study (Table 2). Across plants with positive detections using eDNA, none were found in more than 3 of 6 replicates.

### A framework for monitoring and management actions related to novel aquatic invasive species detection using eDNA metabarcoding data

**Fig. 3:**
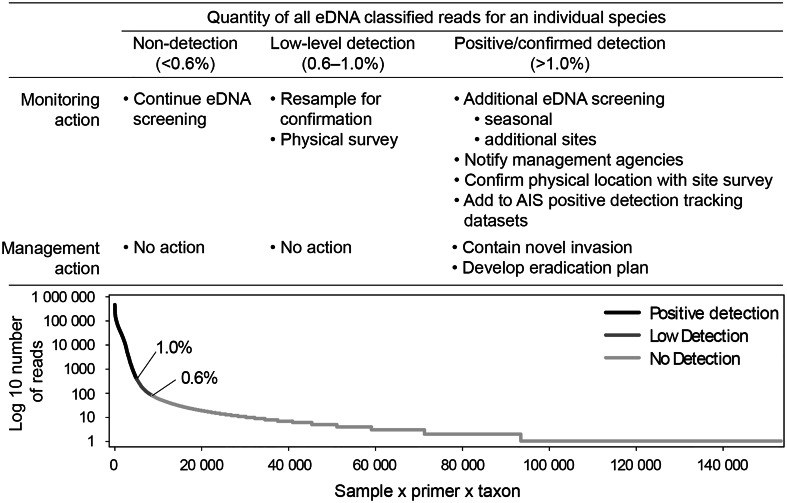
The Aquatic Invasive Species management framework relies on an interpretation of the magnitude of eDNA reads tallied per taxa per sample in the overall classified read count (Log 10 number of reads). Metabarcoding data has a long tail of randomly distributed reads due to index hopping (see “Defining a Detection” section of the Discussion for an explanation of why thresholds of 0.6% and 1.0% were established). The reads tallied for each sample/primer/taxon combination in the lower 0.6% of the dataset are dropped, the detections from 0.6–1% require validation, and detections above the 1% threshold are considered positive. By differentiating between a positive detection and a low detection, this management framework offers a threshold below which validation of the detection should occur before additional management actions are triggered, retaining the sensitivity of the method without reacting to a potential false positive.

Successful integration of eDNA metabarcoding within management frameworks that must also balance other factors such as stakeholder priorities, and invasion risk, is needed to avoid expensive management responses that are initiated by spurious findings such as false positives from non-mediated index swaps. We developed a framework to trigger monitoring or management actions based on the magnitude of eDNA metabarcoding detection results. We reason that higher read counts likely reflect greater confidence of species presence or establishment. Details of the detection-level thresholds are described in the “Defining a Detection” section of the Discussion. Detections with low numbers of reads are handled differently than detections with high numbers of reads so that we can mediate for index swapping without losing the sensitivity of the method. In the framework (Fig. 3), the first step is metabarcode screening for species of interest. Those species not detected remain as part of a multi-species eDNA screening effort. For low detections, the management framework recommends re-sampling for eDNA, and completing traditional surveys to confirm the eDNA finding. Repeated detections or visual confirmation would move the detection of that species from the low-level into the positive/confirmed detection category. Species with a positive (high read count), or confirmed population presence, require additional sites to be selected for screening and traditional surveys to determine the location of the invasion for targeted eradication action. Management agencies must be notified at this point, and the AIS detection needs to be added to tracking datasets. At this stage, management actions are prudent to contain the novel invasion and initiate the development of eradication plans (Fig. 3).

### Positive detection variability over time by water body type

**Fig. 4:**
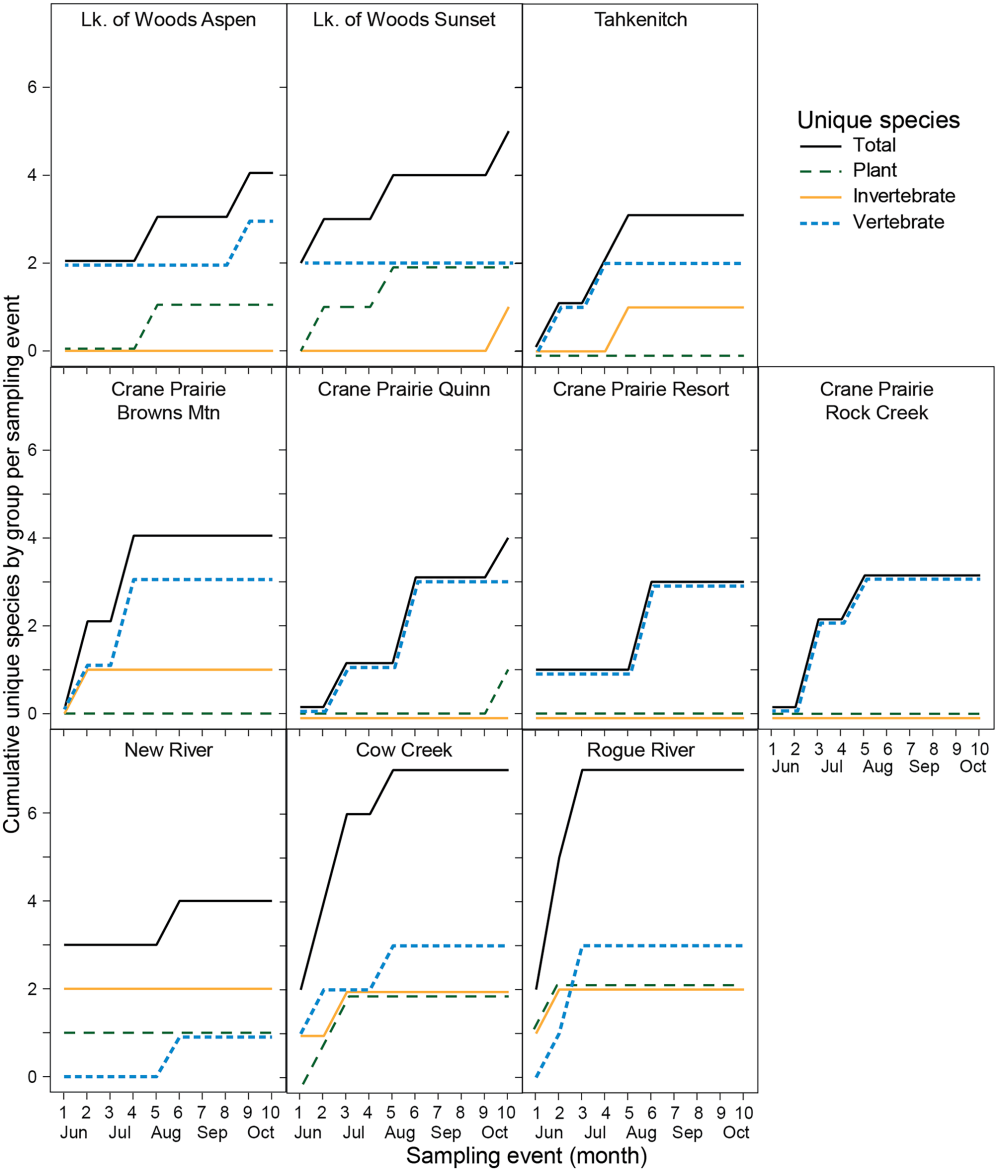
Cumulative species detections varied over time, with rivers often capturing the full diversity of detections by the third sampling event in early July. Lakes and reservoirs saw ongoing additional new detections through the 7th or 8th sampling events in September.

Seasonal changes in species counts differed by water-body type. Reservoirs had the most consistent number of positive vertebrate-species detections across the sampling season (June through October) compared with lakes and rivers. The highest counts of positive vertebrate detections occurred mid-season (August) across water bodies. More positive detections of invertebrates occurred in rivers compared with reservoirs and lakes. The highest number of invertebrate-species detections occurred in June in rivers. No plants had positive detections at reservoir sample sites (although there were low-eDNA detections of several species). The highest counts of positive species detections across taxonomic groups (e.g., vertebrates, invertebrates, and plants) occurred at the beginning of the season in rivers (June), at the end of the season in lakes (October), and mid-season in reservoirs (August; Fig. 4).

#### Results of sampling targeting Dreissena spp. after eDNA-low detection

No detections of either Zebra or Quagga Mussels were found after veliger or eDNA sampling in the 2023 follow-up survey. By the framework we have offered, we would suggest that this species remain as part of the multi-species eDNA screening effort and be catalogued as not observed.

## Discussion

We found that multigene eDNA metabarcoding can identify comprehensive AIS communities, including plants and animals from the same water samples in large water bodies across Oregon, USA (sample sites encompassed both wet/low-elevation and dry/high-elevation environments). This demonstrates that managers can meet multiple management objectives by looking for species across the plant and animal kingdoms sharing the same sample. Overall, we found detections of AIS across more sites and water bodies than was previously documented suggesting silent invasions for some species and greater spread for others. Field sampling showed that different water bodies yielded higher number of species detections at different times of the year, late spring or early summer (June) in rivers, autumn (October) in lakes, and summer (August) in reservoirs. By targeting water bodies with known populations of AIS, we evaluated the effectiveness of eDNA sampling for multiple targets, revealing the sensitivity of eDNA metabarcoding for a multispecies community of AIS. The most comprehensive results were obtained using 6 replicates during late August and early September, but detections varied by taxon, with the most detections for fishes, followed by invertebrates, amphibians, and submerged plants. Terrestrial, emergent riparian plants, had limited detection success on our array. Failure to detect Yellow Flag Iris and Purple Loosestrife could be due to primer failure, DNA shedding or sampling strategy. We did not have Purple Loosestrife in our positive control, but the control results for Yellow Flag Iris indicate that target shifting would be beneficial as the reads could not be identified beyond the genus level for most of the trnH-psbA amplicons. Additional taxonomic and genetic resolution is necessary to distinguish between other plant and animal taxa, including crayfishes.

We integrated our results into a management framework designed to minimize detection error by using a threshold at which to determine low and positive eDNA detection. We demonstrated the usefulness of this framework with our novel low-eDNA detection of a high-interest invasive mussel species (Quagga Mussel) at two of our study sites. Our framework pointed to follow-up with traditional and eDNA sampling to confirm a low-eDNA detection. In our additional sampling, we found no evidence of the presence of this species, allowing for the categorization of this species as currently undetected, avoiding development of expensive management interventions that would have been necessary if a confirmed positive detection had been found.

### Defining a detection

Although the power of next-generation sequencing allows for the extraction of a tremendous amount of information from eDNA samples, it does come with technical and management challenges. As managers begin to incorporate eDNA surveys into protocols and plans, they may choose to use more than one criterion to define a positive eDNA detection as part of a decision-making framework. For example, a threshold of a positive eDNA detection could be set for a given number of replicates to separate a consistent series of strong detections from one or two weak detections (e.g., low detection) and suggest using occupancy modeling to understand detection probabilities. We suggest that as the discussion of eDNA as a management tool continues, it is important to distinguish between the science of eDNA (e.g., methodological sensitivities, limitations) and the implications that are derived from its information (such as AIS presence, e.g.^14^]). In metagenomic datasets, barcode or index swapping is a well-documented phenomenon that occurs on high-throughput sequencers using patterned flow cells such as the Novaseq machine that generated our data. It is thought to affect anywhere from 0.2 to 6% of all data from this platform^27–29^. The fluidigm platform sequences the barcode region using the i7 index, which has been shown to be far less likely to result in index swapping. Costello et al.^29^ found the single i7 swaps rate was 0.6% and the total i7 barcode swap rate (including single i7 swaps, double-indexed swaps, and undetermined (i3 or i7) swaps) was 1.3%. Because barcode swaps are considered random, they show up in multiplexed eDNA data as low number read counts per species forming a long tail in the distribution of the data (Fig. 3).

We found randomly distributed reads of taxa with low number counts in our demultiplexed dataset. We attributed their presence to index swapping or sequencing error in the barcode region of the amplicon. It is difficult to determine where trace eDNA signals begin and potential index swapping ends. To address this problem, we developed a rule that removes portions of the low-read number data tail to eliminate potential false detections that result from sequencing errors and barcode swapping (Fig. 3). The bottom 0.6% (the lowest expected i7 barcode swap rate^29^ of all demultiplexed and classified reads tallied by organism, primer, and sample, was removed from the dataset creating a new ‘non-detection’ cutoff baseline (in this dataset, all read sums < 81). A second threshold at 1% of the data tail was then generated (in this dataset, all read sums = 363) and counts below this threshold but above the 0.6% cutoff were considered to be “low” detections. We chose 1% instead of 1.3% because we do not have dual barcodes and do not need to account for double index swaps. Counts above the 1% data-tail threshold were considered “positive” for that taxon in that sample × primer combination as random barcode swapping should not exceed that 1% threshold. This allowed us to remove the most likely barcode swaps from the dataset, while retaining the sensitivity of the method, and provide for a “low-detection” category and a “positive” category to guide interpretation of results and to inform management actions. While these cutoff thresholds were a good fit for this sequencing run, it should be noted that each sequencing run will have multiple variables, such as number of barcodes, number of reads and overall sequencing run quality, that may affect the data and long tail distribution resulting from barcode swaps. Therefore, the cumulative threshold cutoff percentage may need to be tailored to the particular datasets themselves to ensure removal of sequencing artifacts while retaining sensitivity of the method. In addition to controlling for barcode swaps we also run negative controls to ensure that we do not have contamination on the array further ensuring that the results are not spurious. The only mediation for false negatives is continued sampling efforts.

### Taxonomic variability in detection

We detected plant, invertebrate, and vertebrate taxa across a suite of targeted AIS, highlighting the ability to identify entire AIS communities from one sample, with variable read counts across and within species and replicates over time. Even if they have the same abundance, taxa are expected to have different read counts owing to biological differences (life history, amount of shed eDNA), sample-to-sample variation, patchy distribution of eDNA in the environment, and water mixing, especially in rivers and reservoirs, even in summer when flows are lower. Our results provide evidence that the transport of eDNA in large waters is insufficient to homogenize the signal of species diversity across replicates at a site. Both shedding and decay rates of eDNA vary by taxon and environmental conditions including water temperature, with higher temperatures leading to greater shedding rates and different taxa shedding different types of DNA^32^. In addition, molecular differences such as choice of genetic locus, characteristics of primers, length of target, and GC-content (or guanine-cytosine content) can also affect our ability to detect a species and impart variability in the detection rates. Although our negative control samples did not show contamination, we found the presence of randomly distributed reads as low number counts of taxa in our data set which we attributed to index swapping or sequencing error in the barcode region of the amplicon. It is essential to remove these platform artifacts from a metagenomic data set generated on a patterned flow-cell prior to analysis to avoid false-positive results.

The most consistently identifiable taxa during the sampling season were fishes, probably because they are mobile, which distributes their DNA throughout the water and enhances the likelihood of detection. Invasive invertebrates including New Zealand Mudsnails and crayfish were also consistently found in samples throughout all time periods though they are not detected in every replicate during a given sampling event, probably owing to the patchy nature of eDNA and the mixing of water in large water bodies. Even so, results were consistent enough to suggest they are dependably present and spreading, and that eDNA might be a good technique for early detection of these species. Another eDNA study targeting New Zealand Mudsnail documented the method’s sensitivity, with detection of as few as 1 individual in a sample^33^. This contrasts with emergent plants, which were not detected at any time in the sampling period using these methods, potentially owing to plant die-back affecting results [e.g^33^, or from less effective DNA capture or extraction methods. Bullfrogs were less represented in sampling results throughout the field season than would be expected considering the broad distribution of this species, although they are semi-aquatic as adults and likely move in and out of water frequently, which could account for their patchy detections.

The sensitivity of detections of invasive crayfish species depended on the gene. For example, the universal crayfish primers using the highly conserved 16 S locus were very sensitive, however we found that this locus did not allow us to identify detections to species for closely related *Faxonius* crayfishes. The COI primers were effective at detection in the positive control, and allowed us to identify the population of *Faxonius* spp. at Cow Creek as *F. neglectus* (Ringed Crayfish). It is most likely that the crayfish detected at Cow Creek by the 16 S marker are also *F. neglectus*. The unknown *Faxonius* crayfish detected by the 16 S marker at Rogue River, New River, and Lake of the Woods Sunset cannot be identified to species. However, the low detections from the COI markers at those sites provide some insights into potential identification, but suggest that primer efficiency for this set could be improved for species verification through further investigation or target shifting. COI primers have been used in detections of other crayfishes in large lakes in California, Nevada, and the Great Lakes^34^. Other work suggests that crayfishes may need more genetic and taxonomic research due to undescribed cryptic diversity^35^.

The unknown mussel population discovered at Tahkenitch Lake demonstrates that eDNA can expose the presence of previously unknown or cryptic organisms. Although the closest match for these eDNA sequences was the mussel *Lasmigona costata*, the percentage identification for that match was only 85.5%, a value that would be expected for somewhat distant relatives. The sequences generating that group of reads were isolated from the dataset and further investigated through NCBI Blasts. There was no match in the database to identify this species at the 16 S loci. This species could be an undescribed native species or perhaps an invader, but for now it remains unknown and further research could identify this species.

### Timing of peak detections

Timing of peak species counts (from June to October) differed among water body types, which may track seasonal changes. Some rivers had their highest counts across taxonomic groups in June when discharge was relatively high. A study of 61 sites on a large river show that eDNA species detection rate is positively associated with discharge that facilitates mixing of eDNA throughout the water column^36^. Such an association may be possible in our study, but further research is needed to understand all the mechanisms explaining higher species detections in large rivers in June. For lakes, late in the sampling season in the last weeks of September and into October yielded the highest species counts across taxonomic groups. Another study focusing on fish in lakes detected fishes in thermally stratified water columns until autumn lake turnover, when lake eDNA became more homogenized^37^. Reservoirs reached peak species counts in August, and as they are rivers that have been dammed, they seem to share timing attributes with both lakes and rivers, peaking between those two time periods. In a study in Japan focusing on invasive fish in reservoirs, they found highest species detections in summer and at sites along the shoreline^38^. Across all water body types, we found that late August–early September yielded the highest number of species detections in the June through October growing season, evidence that late summer may be the best time of year to complete field sampling for a monitoring program that includes each of these environments. Although detections can be missed if there are migratory species that occupy these water bodies at other times of the year, detections can be lower for resident animals when activities are reduced, such as when they are not mating^39^, when flows are high^30^, or during cooler times of the year^40^. A study in dune lakes showed high species detection variability owing to species turnover over time, sometimes by only a week, highlighting the importance of sampling more frequently across time in waterbodies with migratory species^41^. Optimal sampling effort relies on timing that maximizes detections of multiple species across taxa in the AIS community, while retaining the ability to detect the least-detectable species. For monitoring program effectiveness, understanding variation in species detections throughout the seasons is critical to interpreting results across the taxa of interest [e.g.^24^.

### Number of replicates

Environmental DNA is known to be patchy in its distribution in water bodies, one factor supporting the importance of replicates at a location to improve detection of species. This patchiness varies among water bodies depending on how mixed the water is. In rivers, the downstream detection of eDNA varies from the source of the DNA based on flow rate, discharge amount, and characteristics of the organism itself (i.e., temporal variability in rivers^42^; spatial variability in rivers^36^; variability of DNA concentrations and patchiness in aquatic environments^43^; variability in shedding rate, life stages, population size^44^. In lakes, water circulation can be locally associated with incoming water sources, or seasonal as with turnover events. The more limited movement of water in lakes and reservoirs likely enhances patchiness of eDNA in those water bodies. In this study, we found that at least 6 replicates of 500 mL per water sample captured detections of most biota, probably owing to the patchiness of eDNA in large water bodies. We often found detections in three or fewer of the six replicates taken (generally with plants and invertebrates), suggesting that higher-replicate sampling produces a higher likelihood of detection [e.g.^18^. In lakes and reservoirs, we found variation among sample sites in detections of both plants and animals, supporting sampling at multiple locations to effectively evaluate the water body. Because of these challenges in using eDNA as a monitoring tool, continued sampling with multiple replicates is the best mediation for potential false negatives. Fortunately, this metabarcoding platform provides multiple attempts to detect AIS from a single sample, and each amplicon is bioinformatically identified thus reducing the risk of off-target false positives that can result from single species qPCR efforts.

### Utility of the monitoring framework

Our findings provided an avenue to test the monitoring framework that we developed for interpreting the read counts for AIS. This is particularly relevant when using eDNA as an early-detection screening tool when the goal is detection of small numbers of individuals before populations become established. The sensitivity of eDNA requires the designation of a threshold for detection levels to avoid the initiation of intensive management responses until presence of the organisms is confirmed^21^. This framework applies to any AIS issue and helps with interpreting the results within a management context, balancing the sensitivity of eDNA with potential management responses. For example, in lakes and reservoirs in North America, Quagga and Zebra Mussel monitoring could follow this framework, which would help to differentiate responses to a low detection versus a positive detection.

To evaluate our framework, we followed through with increased field sampling and additional eDNA sampling when we found a low detection of Quagga Mussels at two of our sites. This species (along with Zebra Mussels) is not conclusively documented as present in Oregon, and a positive detection of either of these species would launch a significant management response (e.g.^13–15^. Our field and additional eDNA sampling did not detect Quagga Mussels at any of our sites, thereby supporting ongoing screening for this species, but precluding development of a management response. Thus, our framework provided a threshold from which to evaluate detection levels for important invasive species, as well as guidelines for when it may be appropriate to follow through with additional sampling and management response.

Follow-up sampling is an important part of the framework for interpreting management response to AIS, including additional eDNA sampling and field methods that target species of interest. e﻿DNA metabarcoding outperforms traditional surveys across methods^16,17^, but it can be unclear how a detection by eDNA translates into measuring the scale of an invasion. It is expensive and inefficient to mount a full management response to a false positive, so developing a protocol for addressing a strong positive versus a low detection can save time, effort, and scarce funds. We suggest that occupancy modeling can offer an understanding of the probability of detection and false positives^18^ and can categorize the detections into positive or low detections relative to the probability of detection.

### Management implications

This study investigates the efficacy of eDNA sampling for multiple species across taxa at once and the optimal sample timing during the year for different types of water bodies. The results of this study have significant management implications, as conventional methods for multispecies surveillance are complicated and costly^45^, hindering AIS management over broad landscapes. Managers can increase efficiency and decrease field-survey expenses by sampling for the eDNA of multiple species in a single sampling event, but interpretation of those results depends upon the time of the year the sample was taken. Aquatic species have more detections from water samples than terrestrial plant species, so more work is needed to evaluate primer efficiency and lab protocols for plants. Until better methods are available, field crews could identify terrestrial invasive plants by visual observation during each sampling occasion. We suggest that at least 6 replicates of 500-mL water samples may be required for detection of biota through eDNA samples, as this number yielded the most diverse species-specific detections in our surveys. Occupancy modeling could identify detection probabilities for different numbers of replicates for each taxon. Taking additional replicates beyond the most commonly collected 1 to 3 samples may add only a negligible additional cost for a field crew in terms of sampling time, but could represent longer laboratory processing time compared with collection of fewer samples. Sample timing and location by water-body type should be considered to increase the probability of detecting particular high-priority species. Although we demonstrated that invasive crayfish can be detected, better libraries with mitochondrial sequence information could be developed to differentiate among species. False-positive detections of harmful aquatic invasive species are a challenging issue associated with the use of eDNA because premature dissemination of those incorrect data could result in unnecessary and expensive responses. However, the management framework described in this paper decreases the potential of false positives to escalate into a management crisis through the use of minimum detection levels. Multiple-species eDNA sampling is an effective way to inventory for a suite of AIS at a relatively low cost, but managers should be cognizant of the target water-body type and species of interest to optimize effectiveness.

## Methods

### eDNA field sampling methods

#### Water sampling

We sampled six water bodies bi-weekly from June through October of 2018 (Fig. 1). These locations were previously identified by federal forest managers as either high-use locations for recreation (making them vulnerable to AIS introductions) or as having known populations of AIS (an important criterion to allow for species-detection testing). Sites chosen were on federal landownerships and were intended to represent the types of large water bodies (lakes, reservoirs, and non-wadeable rivers) currently outside the sampling frame of existing aquatic monitoring programs^46^; Data S1]. Biweekly sampling was assumed to be frequent enough to capture changes in species presence and signal in the water yet allow time for one crew to sample all water bodies.

We conservatively identified documented presence across the entire water body being sampled (Fig. 1), because we could not determine from the literature the exact location an AIS had been found in any specific water body, highlighting the challenge of localized detections. The two lake sites were Tahkenitch Lake (857 ha [347 ac]) on the Oregon Coast and Lake of the Woods (464 ha [188 ac]) near the crest of the Southern Oregon Cascades. Tahkenitch Lake has documented populations of Brazilian Elodea (*Egeria densa)*, Freshwater Golden Clam, Yellow Flag Iris, bullhead, sunfish, and Largemouth Bass. Lake of the Woods has documented populations of Yellow Flag, American Bullfrog, bullhead, and Largemouth Bass. New River is a river/lake system on the southern Oregon Coast, selected for its large population of New Zealand Mudsnail. The two river sites are the Rogue River, with documented populations of Chinese Mystery Snail, Curly-leaf Pondweed (*Potamogeton crispus)*, Purple Loosestrife, Yellow Flag Iris, Ringed Crayfish, American Bullfrog, bullhead, sunfish, and Largemouth Bass; and Cow Creek on the South Umpqua River, with known populations of American Bullfrog, Ringed Crayfish, Purple Loosestrife, bullhead, sunfish, and Smallmouth Bass. Crane Prairie (1384 ha [3420 ac]) in central Oregon was the only reservoir site. Crane Prairie has populations of Eurasian Water-milfoil (*Myriophyllum* sp.*)*, Big-eared Radix, Chinese Mystery Snail, bullhead, sunfish, and Largemouth Bass.

All water bodies were surveyed at locations with high recreational use. River reaches were surveyed at one boat-launch site. Lakes and the reservoir were surveyed at multiple locations including boat launches and campgrounds distributed around the shoreline of the water body. At each survey site, 6 replicate 500-mL water samples were taken from the shore using a Whirl-Pak^®^ and pumped through a 0.45-µm nitrocellulose membrane (https://www.sterlitech.com) with a Mityvac hand pump (http://www.mityvac.com). Greater water volume might have improved our ability to detect AIS in the sample, but we had to balance that with the amount of time available for sampling at each site. To prevent cross-site contamination, we sampled from piers or docks when possible or entered the site downstream from where water samples were taken. Gloves and equipment (bottles, tweezers, waders) were decontaminated with a 50% bleach solution followed by a triple rinse of deionized water. Although we washed with bleach solution to remove DNA from field gear, we also disinfected with Virkon to eliminate any potential spread of disease organisms, fungi, or invasive propagules (decontamination process informed by Appendix D of the Guide to Preventing Aquatic Invasive Species transport by Wildland Fire Operations^47)^. Filters were loosely rolled, then held and transported on ice until stored at − 20 °C within 6 h of collection.

### eDNA laboratory methods

#### Primer development

To identify aquatic invasive species, we used multigene eDNA metabarcoding screening with 48 primers that were either taxon-general or taxon-specific, targeting taxa identified as focal species of interest for federal fisheries biologists (Table 1^18^. We developed primers as described in Hauck et al.^18^ for use on the Fluidigm platform. Briefly, we developed primers for taxon-specific animal taxa for one or more of the following loci: cytochrome C oxidase (COI), cytochrome B (CytB), NADH dehydrogenase 2 (ND2), and NADH dehydrogenase 4 (ND4) and 16 s rDNA. These primers were designed to amplify a number of species at a time and are not species-specific, rather the regions they amplify allow for identification of the amplicon to the species level. We developed plant primers by targeting selected chloroplast loci: trnH-psbA, trnL-trnK, trnL-trnF, trnL intron, or matK based on the success of previous studies^48,49^. In some cases, a single universal forward primer for trnH-psbA was used with different reverse primers to target multiple plant targets. We targeted didymo at the 18 S rDNA locus, and the pathogenic amphibian chytrid fungus *Batrachochytrium dendrobatidis* (*Bd*) at the rDNA internal transcribed 1 spacer (ITS). In addition to taxon-specific primers, we used universal metabarcoding primers targeting 12 S rDNA and 16 s rDNA. These universal primers provide for bycatch of additional non-targeted species, including native species signals, although identifying reads from universal targets to the species level can be challenging in some cases (primer sequences available in Table S1). All Primers were tested in silico for cross-reactivity with Fluidigm CS-tag primers.

#### DNA extraction, amplification, and sequencing

We followed existing laboratory methods for DNA extraction and target amplification^18,25,27^. We extracted, cleaned, and concentrated DNA using the MoBio Power Water kit (Qiagen, Hilden, Germany) and ZymoClean Large Fragment DNA Recovery Kit (Zymo Research, Irvine, CA, USA). This DNA extraction kit has a patented inhibitor-removal step that, along with the addition of bovine serum albumin (BSA) at 0.2 µg µL^−1^ final volume in the amplification step, mitigates PCR inhibition from contaminants found in environmental DNA^50,51^. We adjusted DNA concentrations to 15 ng µL^−1^ (though some samples with low DNA yields had lower concentrations) for subsequent amplification on a Fluidigm Access Array (Fluidigm). We prepared positive controls from genomic DNA extractions of targeted species as follows: a total of 10 ng from each of 34 species was combined with duplex DNA from bacteriophage lambda (New England Biolabs) for a final concentration of 15 ng uL^−1^ (Table S2). We prepared negative controls containing 15 ng µL^−1^ duplex DNA from bacteriophage lambda (see supplemental information Table 1 for species list). We submitted samples on 48-sample plates, with a positive and negative control on each plate.

Target amplification was performed by the University of Illinois at Urbana-Champaign Roy J. Carver Biotechnology Center using a Fluidigm 48.48 Access Array, with FastStart High Fidelity PCR System dNTPack (Roche), and standard 1-step Fluidigm cycling parameters (35 cycles for individual primers, sample indices, and Illumina control sequences) with a modified annealing temperature of 58 °C. The Fluidigm Access Array is a microfluidic platform that uses a 4-primer amplicon tagging scheme in which target-specific primer pairs amplify 48 different targets in combination with sample-specific barcoded primer pairs in 48 different samples. This allows for the simultaneous amplification of barcoded targets in each of the 2,304 individual reaction chambers. Samples were uniquely tagged during amplification with 10 basepair (bp) indices and barcoded amplicons were pooled by equal volumes prior to sequencing on an Illumina NovaSeq 6000 with 250 bp paired-end reads. Sequences were returned from the Roy J. Carver Biotechnology Center demultiplexed by index and by primer with PhiX removed using a custom pipeline they designed to sort Fluidigm data. For this run, they allowed one mismatch in the index, and two mismatches in the primer.

### Bioinformatic analysis

We Joined overlapping read pairs using dbcAmplicons version 0.9.1^52^. For some plant targets, the amplified region exceeded 500 bp and overlapping was not possible. In those instances, only 200 bp of ‘read 1’ sequences were used in subsequent analyses. The primer region was removed on all reads using Trimmomatic 0.39^53^, and a minimum length filter of 150 bp was used on all targets greater than 180 bp. The prepared sequences were then classified to taxon of origin^25^. Briefly, the reads were analyzed using KMA ver. 1.3.9^54^ using a database constructed from the 11 June 2019 NCBI nucleotide database (excluding environmental and artificial sequences^55^, then processed using CCMetagen 1.2.5^56^. KMA generates a consensus sequence using the McNemar test which smooths out potential sequencing errors in amplicons prior to scoring statistics for template identity. The CC-Metagen classification similarity thresholds were optimized through trial-and-error analysis of positive control data. We found the following settings best fit this data set: class = 80.91, order = 81.21, family = 88.3, genus = 96.2, and species = 97.8. For each sample x primer combination, the number of reads for each taxon was counted and summed and compiled to understand spatial patterns (Data S2) and temporal patterns (Data S3).

### Veliger sampling

#### Field sampling methods

We collected a series of 4x vertical plankton-tow samples on 6, 12, and 13 July 2023 from a longitudinal transect along the longest axis in each of Lake of the Woods (4 samples) and Crane Prairie Reservoir (4 samples). For river samples, we towed the plankton net through slow-moving Cow Creek (1 sample) or held the net in place for a known amount of time at fast-moving Rogue River (1 sample). These 10 veliger samples were allowed to slowly filter through the 10-um mesh filter basket at the base of the plankton net. We stored them in Nalgene bottles in an ice-filled cooler in the dark until returned to the laboratory where they were refrigerated until they could be further filtered through 0.7-µm GF/F filters, stored in scintillation vials with 70% ethanol, and returned to refrigerated storage until they were evaluated by microscopy^57–59^.

#### Laboratory methods

We used cross-polarized light microscopy (CPLM) to evaluate the ten veliger samples taken at our study sites in summer 2023. Cross-polarized light is used to evaluate bivalve larvae that are birefringent. Birefringence is an optical property of a material having a refractive index that depends on the polarization and propagation direction of light^57,60^. Sample material was gently scraped from the GF/F filters into a petri dish and evaluated with CPLM and standard enumeration methods^59^.

## Supplementary Information

Below is the link to the electronic supplementary material.

Supplementary Material 1

## Data Availability

Primers used for bioinformatics analysis available in Supplementary Data Table 1. All raw sequence reads from this dataset are accessible to the public, they have been uploaded into the Sequence Read Archive (SRA) at the National Center for Biotechnology Information (NCBI) and can be accessed by searching for BioProject Accession Number PRJNA1100618 (https://www.ncbi.nlm.nih.gov/bioproject/PRJNA1100618).

## References

[1] Roper, B. B., Capurso, J. M., Peroz, Y. & Young, M. K. Conservation of aquatic biodiversity in the context of Multiple- use management on National forest system lands. *Fisheries* **43** (9), 396–405 (2018).

[2] Reid, A. J. et al. Emerging threats and persistent conservation challenges for freshwater biodiversity. *Biol. Rev.* **94**, 849–873 (2019). 30467930 10.1111/brv.12480

[3] Garcia-Moreno, J. et al. Sustaining freshwater biodiversity in the Anthropocene. In *The Global Water System in the Anthropocene* 247–270 (Springer Science and Business: Berlin/Heidelberg, Germany, (2014).

[4] Havel, J. E., Kovalenko, K. E., Thomaz, S. M. & Amalfitano, S. Kats, L. B. Aquatic invasive species: challenges for the future. *Hydrobiologia* **750**, 147–170 (2015). 32214452 10.1007/s10750-014-2166-0 PMC7087615

[5] Dudgeon, D. et al. Freshwater biodiversity: importance, threats, status and conservation challenges. *Biol. Rev.* **81**, 163–182 (2006). 16336747 10.1017/S1464793105006950

[6] Rahel, F. J. & Olden, J. D. Assessing the effects of climate change on aquatic invasive species. *Conserv. Biol.* **22** (3), 521–533 (2008). 18577081 10.1111/j.1523-1739.2008.00950.x

[7] Vitousek, P. M., D’Antonio, C. M., Loope, L. L., Rejmanek, M. & Westbrooks, R. Introduced species: a significant component of human caused global change. *New. Z. J. Ecol.* **21**, 1–16 (1997).

[8] Pimentel, D., Lach, L., Zuniga, R. & Morrison, D. Environmental and economic costs of nonindigenous species in the unites States. *BioScience* **50**, 53–65 (2000).

[9] Crowl, T. A., Crist, T. O., Parmenter, R. R., Belovsky, G. & Lugo, A. E. The spread of invasive species and infectious disease as drivers of ecosystem change. *Front. Ecol. Environ.* **6**, 238–246 (2008).

[10] Tickner, D. et al. Bending the curve of global freshwater biodiversity loss: an emergency recovery plan. *BioScience* **70** (4), 330–342 (2020). 32284631 10.1093/biosci/biaa002 PMC7138689

[11] Johnson, L. E., Ricciardi, A. & Carlton, J. T. Overland dispersal of aquatic invasive species: a risk assessment of transient recreational boating. *Ecol. Appl.* **11**, 1789–1799 (2001).

[12] Crooks, J. A. & Soulé, M. E. Lag times in population explosions of invasive species: causes and implications. In Invasive Species and Biodiversity Management (eds Sandlund, O. T., Schei, P. J. & Viken) 103–125 (Kluwer Academic, (1999).

[13] Blackman, R. C. et al. Targeted and passive environmental DNA approaches outperform established methods for detection of Quagga mussels, *Dreissena rostriformis bugensis* in flowing water. *Ecol. Evol.* **10**, 13248–13259 (2020). 33304534 10.1002/ece3.6921 PMC7713958

[14] Sepulveda, A. J. et al. Using structured decision making to evaluate potential management responses to detection of dreissenid mussel (*Dreissena* spp.) environmental DNA. *Manage. Biol. Invasions*. **13** (2), 344–368 (2022).

[15] Amberg, J. J., Merkes, C. M., Stott, W., Rees, C. B. & Erickson, R. A. Environmental DNA as a tool to help inform zebra mussel, *Dreissena polymorpha*, management in inland lakes. *Manage. Biol. Invasions*. **10**, 96–110 (2019).

[16] Taberlet, P., Coissac, E., Pompanon, F., Brochmann, C. & Willerslev, E. Towards next-generation biodiversity assessment using DNA metabarcoding. *Mol. Ecol.* **21**, 2045–2050 (2012). 22486824 10.1111/j.1365-294X.2012.05470.x

[17] Valentini, A. et al. Next-generation monitoring of aquatic biodiversity using environmental DNA metabarcoding. *Mol. Ecol.* **25** (4), 929–942 (2016). 26479867 10.1111/mec.13428

[18] Hauck, L. L., Weitemier, K. A., Penaluna, B. E., Garcia, T. S. & Cronn, R. Casting a broader net: using microfluidic metagenomics to capture aquatic biodiversity data from diverse taxonomic targets. *Environ. DNA*. **1**, 251–267 (2019).

[19] Gold, Z. et al. eDNA metabarcoding bioassessment of endangered Fairy shrimp (*Branchinecta* spp). *Conserv. Genet. Resour.* **12**, 685–690 (2020).

[20] Zou, K. et al. eDNA metabarcoding as a promising conservation tool for monitoring fish diversity in a coastal wetland of the Pearl river estuary compared to bottom trawling. *Sci. Total Environ.* **702**, 134704 (2020). 31726353 10.1016/j.scitotenv.2019.134704

[21] Fahner, N. A., Shokralla, S., Baird, D. J. & Hajibabaei, M. Large-scale monitoring of plants through environmental DNA metabarcoding of soil: recovery, resolution, and annotation of four DNA markers. *PLOS ONE*. **11**, e0157505 (2016). 27310720 10.1371/journal.pone.0157505 PMC4911152

[22] Coghlan, S. A., Shafer, C. B. A. & Freeland, J. R. Development of an environmental DNA metabarcoding assay for aquatic vascular plant communities. *Environ. DNA*. **3**, 372–387 (2021).

[23] Palacios Mejia, M. et al. The utility of environmental DNA from sediment and water samples for recovery of observed plant and animal species from four Mojave desert springs. *Environ. DNA*. **3** (1), 214–230 (2021).

[24] Rounds, C. I. et al. Aquatic invasive species exhibit contrasting seasonal detectability patterns based on environmental DNA: implications for monitoring. *Freshw. Biol.* **69** (10), 1479–1493 (2024).

[25] Hauck, L. L. et al. Molecular identity crisis: environmental DNA metabarcoding meets traditional taxonomy-assessing biodiversity and freshwater mussel populations (Unionidae) in Alabama. *PeerJ* 11, e15127 (2023). 10.7717/peerj.15127 PMC10078462 37033728

[26] Weitemier, K. A. et al. Estimating the genetic diversity of Pacific salmon and trout using multigene eDNA metabarcoding. *Mol. Ecol.* **30** (20), 4970–4990 (2021). 33594756 10.1111/mec.15811 PMC8597136

[27] Penaluna, B. E., Cronn, R., Hauck, L., Weitemier, K. A. & Garcia, T. S. Uncovering the hidden biodiversity of streams at the upper distribution limit of fish. *J. Biogeogr.* **50**, 1151–1162 (2023).

[28] Kelly, R. et al. Toward a National eDNA strategy for the united States. *Environ. DNA*. **6** (1), e432. 2024 (2024).

[29] Costello, M. et al. Characterization and remediation of sample index swaps by non-redundant dual indexing on massively parallel sequencing platforms. *BMC Genom.* **19**, 322 (2018). 10.1186/s12864-018-4703-0 PMC5941783 29739332

[30] Curtis, A. N., Tiemann, J. S., Douglass, S. A., Davis, M. A. & Larson, E. R. High stream flows dilute environmental DNA (eDNA) concentrations and reduce detectability. *Divers. Distrib.* **27** (10), 1918–1931 (2021).

[31] Jerde, C. L. Can we manage fisheries with the inherent uncertainty from eDNA? *J. Fish Biol.* **98** (2), 341–353 (2021). 31769024 10.1111/jfb.14218

[32] Andruszkiewicz Allan, E., Zhang, W. G., Lavery, A. C. & Govindarajan, A. F. Environmental DNA shedding and decay rates from diverse animal forms and thermal regimes. *Environ. DNA*. **3** (2), 492–514 (2021).

[33] Kuehne, L. M., Ostberg, C. O., Chase, D. M., Duda, J. J. & Olden, J. D. Use of environmental DNA to detect the invasive aquatic plants *Myriophyllum spicatum* and *Egeria densa* in lakes. *Freshw. Sci.* **39** (3), 521–533 (2020).

[34] Larson, E. R. et al. Environmental DNA (eDNA) detects the invasive crayfishes orconectes Rusticus and Pacifastacus Leniusculus in large lakes of North America. *Hydrobiologia* **800**, 173–185. 10.1007/s10750-017-3210-7 (2017).

[35] Allison, P. F. Jr., Pickich, E. T., Barnett, Z. C. & Garrick, R. C. DNA barcoding is currently unreliable for species identification in most crayfishes. *Ecol. Evol.* **14** (7), e70050 (2024). 39041008 10.1002/ece3.70050 PMC11260883

[36] Mächler, E. et al. Assessing different components of diversity across a river network using eDNA. *Environ. DNA*. **1**, 290–301 (2019).

[37] Littlefair, J. E., Hrenchuk, L. E., Blanchfield, P. J., Rennie, M. D. & Cristescu, M. E. Thermal stratification and fish thermal preference explain vertical eDNA distributions in lakes. *Mol. Ecol.* **30**, 3083–3096. 10.1111/mec.15623 (2021). 32888228 10.1111/mec.15623

[38] Jo, T. et al. Utility of environmental DNA analysis for effective monitoring of invasive fish species in reservoirs. *Ecosphere* **12** (6), e03643 (2021).

[39] Spear, S. F., Groves, J. D., Williams, L. A. & Waits, L. P. Using environmental DNA methods to improve detectability in a hellbender (*Cryptobranchus alleganiensis*) monitoring program. *Biol. Conserv.* **183**, 38–45 (2015).

[40] De Souza, L. S., Godwin, J. C., Renshaw, M. A., Larson, E. & Environmental,. DNA (eDNA) detection probability is influenced by seasonal activity of organisms. *PLoS ONE* **11**(10), e0165273 (2016). 27776150 10.1371/journal.pone.0165273 PMC5077074

[41] Beentjes, K. K., Speksnijder, A. G., Schilthuizen, M., Hoogeveen, M. & van Der Hoorn, B. B. The effects of Spatial and Temporal replicate sampling on eDNA metabarcoding. *PeerJ* **7**, e7335 (2019). 31388472 10.7717/peerj.7335 PMC6662575

[42] Milhau, T. et al. Seasonal dynamics of riverine fish communities using eDNA. *J. Fish Biol.* **98**, 387–398 (2019). 31674010 10.1111/jfb.14190

[43] Furlan, E. M., Gleeson, D., Hardy, C. M. & Duncan, R. P. A framework for estimating the sensitivity of eDNA surveys. *Mol. Ecol. Resour.* **16**, 641–654 (2015). 26536842 10.1111/1755-0998.12483

[44] Coble, A. A. et al. eDNA as a tool for identifying freshwater species in sustainable forestry: A critical review and potential future applications. *Sci. Total Environ.* **649**, 1157–1170 (2019). 30308887 10.1016/j.scitotenv.2018.08.370

[45] Evans, N. T. et al. Fish community assessment with eDNA metabarcoding: effects of sampling design and bioinformatic filtering. *Can. J. Fish. Aquat. Sci.* **74** (9), 1362–1374. 10.1139/cjfas-2016-0306 (2017).

[46] Flitcroft, R. L. et al. Pilot study for multiple aquatic invasive species monitoring. *Stream Notes*. **August 2018**, 6–10 (2018).

[47] Invasive Species Subcommittee (ISSC). Guide to preventing aquatic invasive species transport by wildland fire operations. *Natl. Wildfire Coord. Group. PMS* 444 (2017).

[48] Scriver, M., Marinich, A., Wilson, C. & Freeland, J. Development of species-specific environmental DNA (eDNA) markers for invasive aquatic plants. *Aquat. Bot.* **122**, 27–31 (2015).

[49] Mallott, E. K., Garber, P. A. & Malhi, R. S. TrnL outperforms RbcL as a DNA metabarcoding marker when compared with the observed plant component of the diet of wild white-faced capuchins (*Cebus capucinus*, Primates). *PLoS ONE*. **13** (6), e0199556. 10.1371/journal.pone.0199556 (2018). 29944686 10.1371/journal.pone.0199556 PMC6019260

[50] Romanowski, G., Lorenz, M. G. & Wackernagel, W. Use of polymerase chain reaction and electroporation of *Escherichia coli* to monitor the persistence of extracellular plasmid DNA introduced into natural soils. *Appl. Environ. Microbiol.* **59** (10), 3438–3446 (1993). 8250566 10.1128/aem.59.10.3438-3446.1993 PMC182471

[51] Widmer, F., Seidler, R. J. & Watrud, L. S. Sensitive detection of Transgenic plant marker gene persistence in soil microcosms. *Mol. Ecol.* **5** (5), 603–613. 10.1111/j.1365-294X.1996.tb00356.x (1996).

[52] Settles, M., Gerritsen, A. & dbcAmplicons (Version 0.9.1). Accessed March 11, (2019). https://github.com/msettles/dbcAmplicons/docs/manual/DBC_ampliconsUserManual.pdf at master · msettles/dbcAmplicons · GitHub (2014).

[53] Bolger, A. M., Lohse, M., Usadel, B. & Trimmomatic A flexible trimmer for illumina sequence data. *Bioinformatics* **30**, 2114–2120 (2014). 24695404 10.1093/bioinformatics/btu170 PMC4103590

[54] Clausen, P. T., Aarestrup, F. M. & Lund, O. Rapid and precise alignment of Raw reads against redundant databases with KMA. *BMC Bioinform.* **19**, 307 (2018). 10.1186/s12859-018-2336-6 PMC6116485 30157759

[55] Marcelino, V. R. Indexed reference databases for KMA and CCMetagen. [Data file]. The University of Sydney 10.25910/5cc7cd40fca8e (2019).

[56] Marcelino, V. R. et al. CCMetagen: comprehensive and accurate identification of eukaryotes and prokaryotes in metagenomic data. *Genome Biol.* **21** (1), 1–5 (2020). 10.1186/s13059-020-02014-2 PMC7189439 32345331

[57] O’Meara, S., Hosler, D., Brenimer, S. & Pucherelli, S. F. Effect of pH, ethanol concentration, and temperature on detection of Quagga mussel (*Dreissena bugensis*) birefringence. *Manage. Biol. Invasions*. **4** (2), 135–138. 10.3391/mbi.2013.4.2.06 (2013).

[58] Karatayev, A. Y. & Burlakova, L. E. What we know and don’t know about the invasive zebra (Dreissena polymorpha) and Quagga (Dreissena rostriformis bugensis) mussels. *Hydrobiologia*10.1007/s10750-022-04950-5 (2022). 36258710 10.1007/s10750-022-04950-5 PMC9559155

[59] Wetzel, R. G., Likens, G. E. & Collection, Enumeration, and biomass of zooplankton. In *Limnological Analyses* (eds Wetzel, R. G. & Likens, G. E.) 167–178 (Springer, 1991). 10.1007/978-1-4757-4098-1_11.

[60] Frischer, M. E., Kelly, K. L. & Nierzwicki-Bauer, S. A. Accuracy and reliability of *Dreissena* spp. Larvae detection by cross-polarized light microscopy, imaging flow cytometry, and polymerase chain reaction assays. *Lake Reserv. Manag.* **28**, 265–276 (2012).

