# Supplementary material for: Multi-species eDNA as a screening tool to facilitate early detection and eradication of aquatic invasive species in large water bodies
Source: Sci Rep. 2025 Sep 29;15:33615. doi: 10.1038/s41598-025-19083-7 (PMC12480646; doi:10.1038/s41598-025-19083-7)

Supplementary Information

**Multi-species eDNA as a screening tool to facilitate early detection and eradication of aquatic invasive species in large water bodies**

Rebecca L Flitcroft^1^*, Brooke E. Penaluna^1^, Laura L. Hauck^1^, Jay W. Munyon^1^, James M. Capurso^2^

^1^USDA Forest Service, Pacific Northwest Research Station, Corvallis, OR 97331

^2^USDA Forest Service, Pacific Northwest Regional Office, Portland, OR 97204

*Corresponding author: [rebecca.flitcroft@usda.gov](mailto:rebecca.flitcroft@usda.gov)

Supplementary Table S1

Supplementary Table S2

Supplementary Data S1

Supplementary Data S2

Supplementary Data S3

Table S1. Primer sequences corresponding to focal species of interest included: 20 primers targeting invasive animal species, 18 primers targeting invasive plants and algae, 2 primer sets targeting pathogens, and 7 universal metabarcoding primers targeting a wide range of organisms.


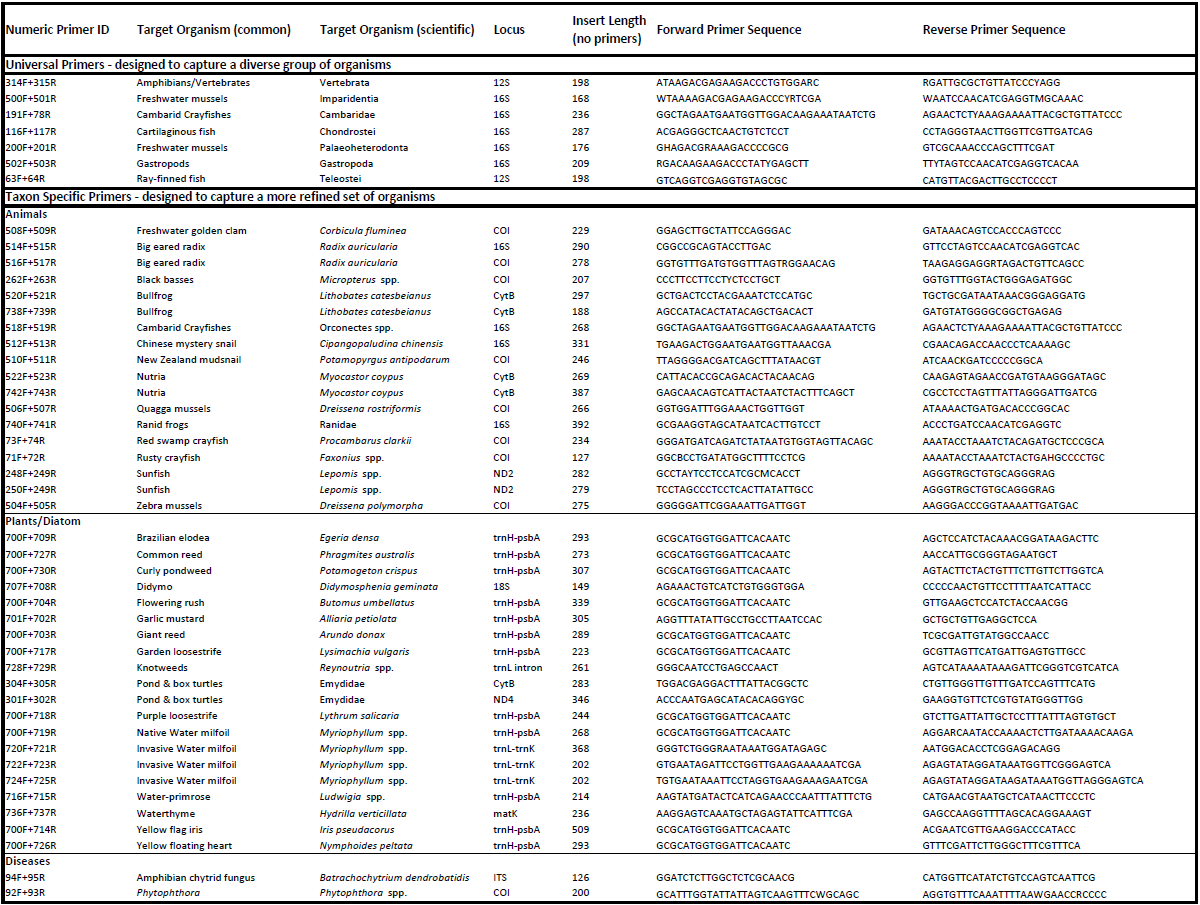


Table S2. Genomic DNA of species put in the positive control sample. One positive control and is put on each plate.

| **Genomic DNA Samples in the Positive Control:** | |
| --- | --- |
| **Common Name** | **Species** |
| Sturgeon | *Acipenser transmontanus* |
| Water-hyssop | *Bacopa caroliniana* |
| BD/Chytrid | *Batrachochytrium dendrobatidis* |
| Freshwater Golden Clam | *Corbicula fluminea* |
| Zebra Mussel | *Dreissena polymorpha* |
| Quagga Mussel | *Dreissena rostriformis bugensis* |
| Brazilian waterweed | *Egeria densa* |
| Yellow-flag Iris | *Iris pseudacorus* |
| water primrose-willows | *Ludwiga sediodes* |
| [Large-flower primrose-willow](https://gcc02.safelinks.protection.outlook.com/?url=https%3A%2F%2Fwww.cabi.org%2Fisc%2Fdatasheet%2F109148&data=02%7C01%7C%7Cfd32067a8a0e43c414dd08d731685b7e%7Ced5b36e701ee4ebc867ee03cfa0d4697%7C0%7C0%7C637032196591866399&sdata=sz%2F71RolSNUw20oEEoqqvgHiklCn%2FLx9jQQk2bXkj5g%3D&reserved=0) | *Ludwigia grandiflora* |
| [Large-flower primrose-willow](https://gcc02.safelinks.protection.outlook.com/?url=https%3A%2F%2Fwww.cabi.org%2Fisc%2Fdatasheet%2F109148&data=02%7C01%7C%7Cfd32067a8a0e43c414dd08d731685b7e%7Ced5b36e701ee4ebc867ee03cfa0d4697%7C0%7C0%7C637032196591866399&sdata=sz%2F71RolSNUw20oEEoqqvgHiklCn%2FLx9jQQk2bXkj5g%3D&reserved=0) | *Ludwigia hexapetela* |
| water primrose-willows | *Ludwigia peploides* |
| water primrose-willows | *Ludwigia pervensis* |
| Largemouth Bass | *Micropterus nigricins* |
| watermilfoils | *Myriophyllim aquaticus* |
| Parrot-feather milfoil | *Myriophyllim aquaticum* |
| watermilfoils | *Myriophyllim hippuroides* |
| watermilfoils | *Myriophyllim quitense* |
| watermilfoils | *Myriophyllim sibiricum* |
| Eurasian milfoil | *Myriophyllim spicatum* |
| "Myrio Red" milfoil cultivar | *Myriophyllum heterophyllum* |
| watermilfoils | *Myriophyllim spicatum* |
| "Snowflake Fringe" cultivar | *Nymphoides geminata* |
| Alabama Hickory Nut | *Obovaria unicolor* |
| cutthroat trout (for 16S) | *Oncorhynchus clarkii* spp*.* |
| Ringed Crayfish | *Faxonius neglectus* |
| Rusty Crayfish | *Faxonius rusticus* |
| Common reed | *Phragmites australis* |
| Phytophthora lateralis | *Phytophthora lateralis* |
| Red Swamp Crayfish | *Procambarus clarkii* |
| Big Eared Raddix | *Radix auricularia* |
| Red Legged Frog | *Rana aurora* |
| Bull Frog | *Lithobates catesbeianus* |
| Red Eared Slider | *Trachymys scripta* |

**Dataset legends**

Dataset S1. Snapshot of data file with geospatial coordinates of sampling sites that was used to create Figure 2. Site Name is the name of the site including the name of the water body and the place (often the boat ramp or dock) on that water body where sample was taken. Site is the three-letter code for the site name. Waterbody Type is either lake, reservoir, or river that corresponds with the site. X is the X-coordinate and Y is the Y-coordinate for the geospatial information using WGS84.


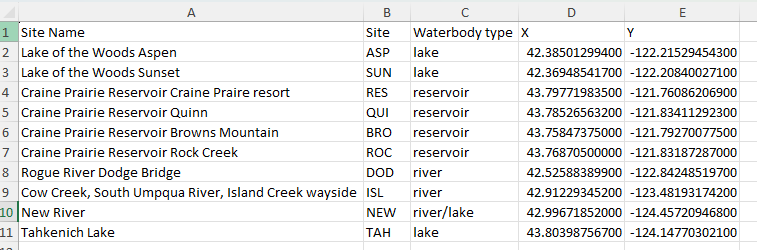


Dataset S2. Snapshot of data file used to understand spatial patterns and to create Figure 3. Primer is the primer name by our lab group for what we targeted. Site is the three-letter code for the site name. Timepoint is which of the 10 sampling time periods that we sampled from 1 to 10. Replicate is replicate number from 1 to 4. Lab ID# is the number assigned to that sample by our lab. Sample is the specific name of that sample, including the Lab ID#. Barcode incorporates the barcode target. Sample ID is the specific sample code coming in from the field. Number_Reads are the number of reads for that sample and species. Domain is the biological level of Domain detected for that specific sample and primer given our data pipeline and library. Kingdom is the biological level of Kingdom detected for that specific sample and primer given our data pipeline and library. Phylum is the biological level of Phlyum detected for that specific sample given our data pipeline and library. Class is the biological level of Class detected for that specific sample and primer given our data pipeline and library. Order is the is the biological level of Order detected for that specific sample and primer given our data pipeline and library. Family is the is the biological level of Family detected for that specific sample and primer given our data pipeline and library. Genus is the is the biological level of Genus detected for that specific sample and primer given our data pipeline and library. Species is the is the biological level of Species detected for that specific sample and primer given our data pipeline and library.


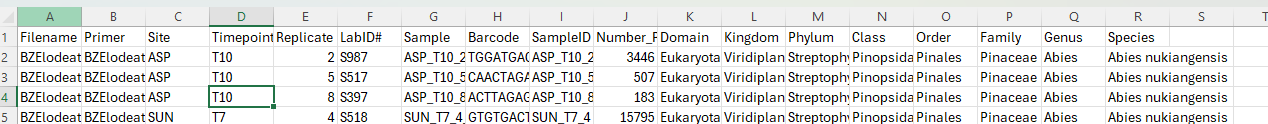


Dataset S3. Snapshot of data file used to understand temporal patterns and to create Figure 4. Site is the three-letter code for the site name. Timepoint is which of the 10 sampling time periods that we sampled from 1 to 10. Site Name is the name of the site including the name of the water body and the place (often the boat ramp or dock) on that water body where sample was taken. Waterbody Type is either lake, reservoir, or river that corresponds with the site. Species Interest is the number of species targets with detections in this study. Vertebrate is the number of targeted vertebrate species with detections in this study. Plant is the number of targeted plant species with detections in this study. VertUnique is the number of targeted vertebrate species that were unique to that site during that timepoint. InvertUnique is the number of targeted invertebrate species that were unique to that site during that timepoint. PlantUnique is the number of targeted plant species that were unique to that site during that timepoint. TotalUnique are the number of targeted species for vertebrates, invertebrates, and plants that were unique to that site across timepoints. TotUnVert are the number of targeted species for vertebrates that were unique to that site across all timepoints. TotUnInvert are the number of targeted species for invertebrates that were unique to that site across all timepoints. TotUnPlant are the number of targeted plant species that were unique to that site across all timepoints. Column 1 to Column 14 list the name of the species identified at that site and timepoint for vertebrates, invertebrates, or plants.


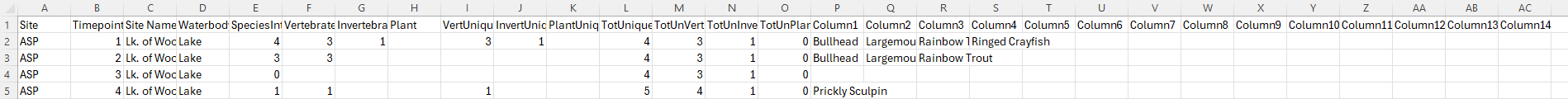

Supplement: Supplementary file 1 — Supplementary Material 1 [file 41598_2025_19083_MOESM1_ESM.docx]
